# Genetic Heterogeneity in Alzheimer Disease and Implications for Treatment Strategies

**DOI:** 10.1007/s11910-014-0499-8

**Published:** 2014-09-14

**Authors:** John M. Ringman, Alison Goate, Colin L. Masters, Nigel J. Cairns, Adrian Danek, Neill Graff-Radford, Bernardino Ghetti, John C. Morris, Dominantly Inherited Alzheimer Network

**Affiliations:** 1 Mary S. Easton Center for Alzheimer’s Disease Research, David Geffen School of Medicine at University of California, Los Angeles, 10911 Weyburn Ave., #200, Los Angeles, 90095-7226 CA USA; 2 Department of Neurology, Washington University School of Medicine, 660 S. Euclid Ave., St Louis, 63110 MO USA; 3 The Florey Institute, University of Melbourne, Level 5, Kenneth Myer Building 30 Royal Parade, Parkville, 3010 VIC Australia; 4 Neurologische Klinik Ludwig-Maximilians-Universität Munich and German Center for Neurodegenerative Diseases, Marchioninstr. 15, Munich, D-81377 Germany; 5 Mayo Clinic, 4500 San Pablo Road, Jacksonville, 32224 FL USA; 6 Department of Pathology and Labortory Medicine, Indiana University School of Medicine, 635 Barnhill Drive, MSA138, Indianapolis, 46202 IN USA; 7 Mary S. Easton Center for Alzheimer’s Disease Research, UCLA Department of Neurology, 10911 Weyburn Ave., #200, Los Angeles, CA 90095 -7226 USA

**Keywords:** Alzheimer’s disease, Genetic, Heterogeneity, Presenilin, Amyloid precursor protein, Apolipoprotein E

## Abstract

Since the original publication describing the illness in 1907, the genetic understanding of Alzheimer’s disease (AD) has advanced such that it is now clear that it is a genetically heterogeneous condition, the subtypes of which may not uniformly respond to a given intervention. It is therefore critical to characterize the clinical and preclinical stages of AD subtypes, including the rare autosomal dominant forms caused by known mutations in the *PSEN1*, *APP*, and *PSEN2* genes that are being studied in the Dominantly Inherited Alzheimer Network study and its associated secondary prevention trial. Similar efforts are occurring in an extended Colombian family with a *PSEN1* mutation, in *APOE* ε4 homozygotes, and in Down syndrome. Despite commonalities in the mechanisms producing the AD phenotype, there are also differences that reflect specific genetic origins. Treatment modalities should be chosen and trials designed with these differences in mind. Ideally, the varying pathological cascades involved in the different subtypes of AD should be defined so that both areas of overlap and of distinct differences can be taken into account. At the very least, clinical trials should determine the influence of known genetic factors in post hoc analyses.

## Introduction

Alzheimer’s disease (AD) is often conceptualized as a unitary clinicopathological entity characterized by progressive loss of memory and other changes in cognition and behavior that ultimately affect self-care. The disease is defined neuropathologically by neuronal loss, gliosis, and the abnormal accumulation of amyloid β (Aβ) as extracellular plaques and by intraneuronal accumulations of hyperphosphorylated tau protein as neurofibrillary tangles and dystrophic neurites. For decades after it was first described [1], AD was considered a distinctive “presenile” cause of dementia until attention was brought to its histological commonalities with dementia of late onset [2]. Subsequent advances in our understanding of the clinical, genetic, and neuropathological heterogeneity of AD, however, bring into question its identity as a single entity. Despite this, most of the current clinical trials being performed to test interventions for AD do not discriminate between disease subtypes. This approach will fail to identify treatments that might be preferentially beneficial in subsets of persons with AD. It is thus critical that we have a better understanding of the taxonomy of the conditions grouped as AD. Disease subtypes may be categorized according to their clinical, imaging, histological, or other features. Although lifestyle and environmental risk factors clearly affect the manifestations of AD pathology, the primacy of genetic influences and our increasing understanding of them suggests that categorization by genetic basis should be prioritized in our effort to develop effective interventions.

The heritability of AD is estimated to be between 58 and 79 % [3]. After increasing age, genetic factors play the most important role in the development of the disease. It has long been recognized that forms of AD often have a familial component [4], but only rarely is it clearly inherited in a highly penetrant autosomal dominant fashion. It is has been well established that the inheritance of a vulnerability to AD pathology is complex, and AD is indeed a model of genetic heterogeneity [5]. The inheritance of AD might be considered to be on a spectrum, with highly penetrant mutations causing autosomal dominant familial AD, usually of young onset, at one end and combinations of common variants with small effects contributing to “sporadic” late-onset AD (LOAD) at the other [6]. Additional rare variants with large effects have been identified that contribute to AD risk in LOAD families [7]. Arguably the most relevant genetic risk factor for LOAD is the polymorphic gene encoding apolipoprotein E (ApoE; *APOE*). The risk-conferring allele (ε4), when present in the homozygous state, although not determinant for the development of AD, confers as much as a 15 times increased risk of developing the disease [8].

Variants in genes coding for proteins involved in processing of amyloid precursor protein (APP), transport of lipids and other molecules, the innate immune system and inflammation, endocytosis, and intracellular tracking have all been associated with differential risk of AD. Our understanding of the specific genetic contributors to most of the late-onset cases is still incomplete, but with improvements in our ability to perform DNA sequencing on a large scale, it is likely that we will ultimately be able to disentangle the genetic contribution in any given individual. In this article we will review the evidence for subtyping AD according to genetic origins as a starting point towards a comprehensive understanding of this heterogeneous group of conditions that might collectively be thought of as the Alzheimer’s diseases (AD). We will discuss the unique contributions that are being made through the study of autosomal dominant AD (ADAD) that is being performed in the Dominantly Inherited Alzheimer Network (DIAN).

As the future development of AD can be reliably predicted in persons carrying fully penetrant, autosomal dominantly inherited mutations, study of asymptomatic or minimally symptomatic persons at 50 % risk of inheriting them provides a unique opportunity to characterize clinical, cognitive, imaging, and biochemical changes occurring early in the cascade leading to clinical disease [9]. As these mutations are rare, such studies were performed on relatively small scales at a number of sites worldwide. These studies led to a number of insights into the early changes of the illness, but were limited by the absolute numbers of subjects that could be enrolled as well as the difficulties encountered when making comparisons across cohorts and studies. To facilitate such studies, the National Institute on Aging funded the international DIAN, which is longitudinally assessing persons known to be at risk of inheriting ADAD mutations using a uniform clinical, cognitive, and imaging protocol and plasma and cerebrospinal fluid samples acquired using common procedures [10]. Persons determined not to carry the ADAD mutation that runs in their family serve essentially as controls, allowing the identification of disease-associated changes. As the age of disease onset can also be predicted fairly reliably within a family, the temporal ordering of changes can also be estimated [11•]. The DIAN reached its targeted enrollment of 400 subjects, represented by 80 ADAD mutations, prior to the end of its first grant cycle (Table 1). This has allowed definition of measurable disease markers during the preclinical stages of the illness [12]. Although there may be some limitations to the generalizability of observations in ADAD to other forms of the disease (see later), observations from the DIAN have facilitated the design and execution of trials to prevent this group of diseases.

**Table 1:** Mutations in the presenilin 1, presenilin 2, and amyloid precursor protein genes causing early-onset autosomal dominant Alzheimer’s disease represented in the Dominantly Inherited Alzheimer Network study

*PSEN1* mutations		
Ala79Val	Leu171Pro	Ala246Glu
Cys92Ser	Phe176Val	Ala260Gly
Phe105Leu	Ser178Pro	Ala260Val
Phe105Ser	Glu184Asp	Val261Ile
Deletion intron 4	Ile202Phe	Val261Phe
	Gly206Ala	Pro264Leu
Tyr115Cys	Gly206Val	Arg269His
Tyr115His	Gly209Glu	Leu271Val
Thr116Ile	Gly209Val	Ala275Val
Ser132Ala	Ser212Tyr	Arg278Ile
Asn135Ser	Ile213Leu	Glu280Ala
Asn135Tyr	Gly217Arg	Glu280Gly
Met139Ile	Leu219Pro	Phe283Leu
Met139Val	Gln222His	Tyr288His
Ile143Thr	Leu226Arg	Ser290Cys
Met146Ile	Ile229Phe	Deletion exon 9
Met146Leu	Ser230Asn	Gly378Glu
Met146Val	Met233Leu	Leu392Val
Thr147Ile	Met233Thr	Cys410Tyr
His163Arg	Leu235Val	Ala426Pro
Ser169Leu	Ile238Met	Ala431Glu
Ser170Phe	Thr245Pro	Ile439Val
	Phe176Val	
	Ser178Pro	
	Glu184Asp	
	Ile202Phe	
	Gly206Ala	
	Gly206Val	
	Gly209Glu	
	Gly209Val	
	Ser212Tyr	
	Ile213Leu	
	Gly217Arg	
	Leu219Pro	
	Gln222His	
	Leu226Arg	
	Ile229Phe	
	Ser230Asn	
	Met233Leu	
	Met233Thr	
	Leu235Val	
	Ile238Met	
*PSEN2* mutations		
Arg62His	Ser130Leu	Asn141Ile
*APP* mutations		
Lys670Asn and Met671Leu	Ile716Phe	Val717Phe
Ala692Gly	Val717Ile	Leu723Arg
Glu693Gln	Val717Leu	Leu723Pro
Ile716Val		

## Autosomal dominant forms of AD

In 1907, Alois Alzheimer published a description of the neuropathological changes found in a woman who died aged 55 years with dementia [11•]. Although familial forms of the disease were described as early as the 1930s [4], the first genetic variant to be unequivocally associated with β-amyloidosis was a missense mutation (E693Q) in the *APP* gene described in 1990 [13]; this mutation causes intracerebral hemorrhage and sparse parenchymal diffuse plaques. As cleavage products of APP had previously been identified to be the major components of the angiopathy [14–16] and amyloid plaques [14, 16, 17] associated with AD, an important link between genetics and pathological changes was established. It was subsequently recognized that mutations near the β-secretase (e.g., K670N, M671L) and γ-secretase (e.g., V717I) cleavage sites are associated with increased absolute or relative production of the 42-amino acid Aβ product of APP (Aβ42) [18]. Pathogenic mutations in the most commonly affected gene causing ADAD, presenilin 1 (*PSEN1*) [19], and its homolog, presenilin 2 (*PSEN2*) [20], similarly increase the relative production of Aβ42 [18–20, 21•]. Aβ42 is the major component of the extracellular plaques that define AD, and has an increased propensity to bind with itself and form the β-pleated structures found in these plaques. This convergence of data has driven the unifying “amyloid hypothesis” regarding the origin of AD [22] that has dominated to this day [22]. Further supporting this hypothesis is the fact that the phenotypic and pathological features of ADAD and LOAD can, at least superficially, appear similar. Although the continuity between these forms of AD is striking, when the clinical, neurochemical, and neuropathological associations of *APP* and presenilin mutations are scrutinized, differences from LOAD can be identified.

In addition to the younger age of onset, persons with familial AD mutations, particularly those with *PSEN1* mutations, can have clinical features that are quantitatively or qualitatively atypical of LOAD. Although myoclonus and seizures are not uncommon in LOAD [23], they tend to occur late in the illness, whereas in ADAD they are more frequent and can occur early [24•]. In a comparison between 32 symptomatic familial AD mutation carriers and a cohort of young AD patients without a family history, an increased prevalence of headaches, pseudobulbar affect, and limb spasticity was also observed [24•]. Others have noted that dysarthria can occur as an early feature [25].

Neuropathological studies have also revealed some differences between ADAD and LOAD. One study that assessed Aβ found that the levels of total Aβ and Aβ42, although not Aβ40, were increased in the cortex of persons with ADAD, and that there could be substantial variability in the neuropathological changes in ADAD, even among family members with the same mutation [26]. However, another study found no significant differences in Aβ burden between ADAD and LOAD [27]. In a recent detailed study comparing the brains of ten persons with ADAD with the brains of persons with LOAD, differences in the nature and distribution of pathology were found [28•]. Although there was no difference in the absolute amount of Aβ42, in persons with LOAD Aβ42 levels were higher in cortical areas than in persons with ADAD, and in ADAD they were was higher in subcortical areas (e.g., amygdala, striatum, hypothalamus, thalamus, cerebellum). Tau pathology tended to be greater in ADAD cases in many areas, with the difference being greatest in the striatum. As Aβ42 levels were highly correlated with synaptic markers (PSD95) in LOAD and with measures of APP metabolism (APP and the β-C-terminal fragment, β-CTF) in ADAD, the study authors concluded that this was evidence for synaptic processes driving Aβ42 accumulation in LOAD, whereas in ADAD this was driven by aberrant APP processing. Not surprisingly, the aforementioned atypical features were commoner in ADAD, and were attributed to the disproportionate subcortical pathology seen in these cases. Hippocampal sclerosis is a common comorbid pathological finding in LOAD, and is frequently associated with TAR DNA binding protein 43 pathology and memory deficits [29]. Although this pathology can be found in ADAD, it appears to be less common [30], occuring in 9 % of ADAD cases relative to 29 % in LOAD, suggesting divergent biochemical cascades.

In a study in which the levels of β-secretase and its catalytic products were compared in the brains of persons with *APP* and *PSEN1* mutations and LOAD, higher levels of the C-terminal fragment of APP produced by β-secretase cleavage (β-CTF) were found in ADAD, although the levels of β-secretase itself and its activity were not elevated [31•]. In contrast, in LOAD, lower levels of β-CTF were found despite elevated levels of β-secretase and β-secretase activity. These results suggest that the mechanisms involved in the imbalance of Aβ production and clearance in these forms of AD are distinct. Another study identified similarly increased activity of the endocytic pathway in neurons and endothelia in brains of persons with certain APP mutations and sporadic AD that was not present in persons with *PSEN1* mutations [32], also suggesting divergent disease mechanisms.

Measurement of Aβ metabolism in vivo in humans has also confirmed that Aβ clearance rates are reduced in LOAD, although production rates are normal [33•], whereas persons with *PSEN1* mutations are characterized by increased Aβ synthesis rates [21•]. These observations collectively suggest that, although some therapeutic targets (e.g., Aβ42 itself) are common between these forms of AD, potentially distinct therapeutic targets might be prioritized (e.g., synaptic activity, β-secretase activity, Aβ clearance) in these different forms of AD.

### Amyloid Precursor Protein

Although the role of APP and its derivatives in AD has been the focus of substantial research in the last two decades, its normal physiological function and those of its metabolites are not clear. APP is present in many tissues, and exists as a membrane protein in brain, where there is evidence that it is involved in cell–cell and cell–substrate interaction [34] and gene regulation [35], and has a role in brain development and synaptogenesis. A complete discussion of the physiological functions of APP and its metabolites is not possible here [36]; we will focus on those that suggest a differential role in AD subtypes.

Mutations in *APP* are the second commonest cause of ADAD, representing 32 % of families and 21 % of individuals enrolled in the DIAN observational study. Disease-causing mutations in *APP* include those near the β-secretase cleavage site (amino acids 670–682), near the γ-secretase cleavage site (amino acids 713–724), or within the Aβ sequence (amino acids 692–705) of the protein. A caveat to this observation is that much of the early sequencing of *APP* focused on exons 16 and 17, the exons encoding the Aß sequence. It is therefore possible that mutations outside these regions of *APP* may be identified and associate with AD. Nonetheless, in vitro, mutations near the β-secretase cleavage site cause an overall increase in the amount of total Aβ produced [37], whereas mutations near the γ-secretase cleavage site specifically cause an increase in the amount of Aβ42 produced [38]. Mutations within the Aβ sequence are thought to affect the way Aβ self-aggregates [39] and oligomerizes [40]. Additionally, duplication of *APP* is inherited in an autosomal dominant fashion and causes ADAD [41]. There are therefore diverse ways in which alterations of the *APP* gene can lead to AD, and they can have distinctive clinical and histological features.

Among patients with ADAD, patients with *APP* mutations more often present with typical features of AD, including predominant memory problems and medial temporal lobe atrophy [42]. There is, however, tremendous variability between the age of onset and other phenotypic features among different mutations in the *APP* gene, including distinct amino acid substitutions at the same codon. Suarez-Calvet et al. [43] found that the I716F substitution in *APP*, which causes the youngest onset of disease of any *APP* mutation, had additional effects on γ-secretase cleavage, at least in vitro, relative to two other mutations at the same site (I716V and I716T), possibly accounting for its more aggressive, *PSEN1*-like phenotype.

In addition to parenchymal deposition of Aβ, Aβ can also be deposited in the walls of cerebral arterioles and capillaries (cerebral amyloid angiopathy, CAA). Although it is most often asymptomatic, CAA can predispose to symptomatic intracerebral hemorrhages. The degree to which CAA occurs varies widely between individuals, and the reasons for this are incompletely understood. Although the *APOE* genotype plays a role (see below), other factors are clearly involved. Persons with *APP* mutations may have significant CAA, and it is most evident in persons with mutations within the Aβ sequence in which vascular manifestations can predominate the clinical picture [13, 44, 45]. CAA also commonly occurs in persons with *APP* duplication and in Down syndrome (DS; see below). As Aβ40 is the commonest species of Aβ found in affected blood vessels, it has been speculated that genetic alterations that increase the absolute amount of Aβ rather than increasing the Aβ42/Aβ40 ratio predispose to CAA [46]. The potential presence of CAA has important therapeutic implications as antiamyloid treatments being developed for AD can precipitate intracerebral edema and hemorrhage [47]. As this complication occurs more frequently in *APOE* ε4 carriers [48•], in whom CAA is commoner [49], it is likely that CAA predisposes to these adverse vascular events. The risk of CAA-related complications in the context of trials of antiamyloid therapies is one area in which genetically subtyping AD has already been demonstrated to be of utility.

The development of radiolabeled Aβ-binding agents has facilitated the radiological (using positron emission tomography, PET) visualization of Aβ-specific retention in the human brain, a correlate of cerebral β-amyloidosis. PET–amyloid binding provides further grounds for differentiating genetic subtypes of AD. With the use of the amyloid-binding ligand Pittsburgh compound B, atypical and apparently early deposition of Aβ in the striatum not usually seen in LOAD was first described in carriers of *PSEN1* mutations [50] and then in persons with *APP* mutations [51]. The neuropathological study described above in which higher levels of Aβ42 deposition were found in the striatum in ADAD potentially explains this finding [28•]. Conversely, a positive Pittsburgh compound B signal was not seen in two carriers of the E693G *APP* mutation [52, 53] despite the presence of other biomarker evidence of AD pathology. This mutation occurs within the Aβ sequence and features atypical amyloid plaque morphology [54], suggesting a distinctive plaque organization and consequent distinct PET ligand binding properties.

Down syndrome (DS) is a common neurodevelopmental condition most frequently caused by trisomy of chromosome 21, on which the *APP* gene resides. In addition to the significant lifelong mental, cardiac, skeletal, and other abnormalities that are common in DS, persons with DS inevitably develop AD neuropathological changes by age 50 years [55], and frequently have an associated dementia syndrome. It is likely that these neuropathological changes of AD are driven by the increased levels of APP metabolites that can be measured in the plasma [56] of persons with DS. Trisomy of chromosome 21 also leads to the dysregulation of multiple other genes and inflammation [57], oxidative stress [58], and endocytic abnormalities [59, 60], which therefore may also influence the manifestation of AD pathology in persons with DS. There is substantial heterogeneity of AD in DS which is accentuated by cases of partial trisomy in which the *APP* gene is not triplicated and AD pathology is absent [61] and cases of mosaicism lacking the full DS phenotype in which early-onset AD nonetheless occurs [62]. The design and interpretation of AD treatment studies in DS must take this heterogeneity into account.

### The Presenilins

Mutations in *PSEN1* are the commonest causes of ADAD and mutations in *PSEN2* are the least common causes of ADAD, with 185 and 13 pathogenic mutations, respectively, having been described. Families harboring *PSEN1* mutations account for 62 % of families and 71 % of individuals in the DIAN observational study. *PSEN2* mutations appear to be rare, with four families harboring the same N141I mutation (a founder effect [63]) enrolled in the DIAN study, represented by 30 individuals. Person with *PSEN1* mutations have the youngest age of symptom onset and persons with *PSEN2* mutations have the oldest age of symptom onset [11•]. The atypical features of myoclonus, seizures, and corticospinal tract signs are commonest with *PSEN1* mutations. The age of onset can be as young as the mid-20s [64]. The neuropathological changes associated with *PSEN1* mutations can be typical of LOAD, but can also be quite diverse. One type of lesion, the so-called cotton wool plaques, are large diffuse Aβ plaques typically lacking dystrophic neurites and composed predominantly of Aβ42, that have been reported in some *PSEN1* mutation cases. Aβ deposition has been reported in white matter [65] and in the cerebellum [66]. Variable and often severe degrees of CAA [67] and ectopic neurons have also been described [65], but these changes have also been reported in LOAD. The cause of this variation is unknown, but may relate to the gene defect and consequent disruption of multiple cellular pathways. For example, the presenilins, in addition to their role in γ-secretase cleavage of APP, also play roles in various other cell functions, including calcium regulation [68], development [69], and axonal transport [70], and are associated with biochemical changes in white matter [71]. The effects of pathogenic *PSEN1* mutations on γ-secretase cleavage are varied and can lead to the generation of Aβ species not seen in LOAD [72], the implications of which are yet to be determined. An enhanced understanding of presenilin biology may identify therapeutic targets not applicable to LOAD or other forms of AD.

## Apolipoprotein E

The risk-conferring allele of the ApoE gene (*APOE* ε4) is possibly the most robust genetic risk factor for AD, being present in approximately 50 % of persons with LOAD. Having a copy of the *APOE* ε4 allele reduces the age at which AD symptoms manifest themselves, and having two copies reduces it further still, with many cases of AD with onset before age 65 years being due to *APOE* ε4 homozygosity. ApoE plays a role in lipid transport, and also in inflammation [73] and other physiological processes, so the most critical mechanism through which it confers a higher risk of AD is not entirely clear. ApoE also transports Aβ, and it has been shown that the proteins encoded by the different *APOE* polymorphisms have differential effects on this clearance. ApoE ε4 is less effective at clearing Aβ than is ApoE of the ε2 or ε3 allele, and it may be through this effect that the increased risk of AD and CAA [74, 75] is enhanced [76].

The clinical phenotype of *APOE* ε4-related AD appears to be that of the “typical” amnestic presentation [77]. Consistent with this observation, *APOE* ε4 carriers demonstrate more atrophic changes in the CA1 region of the hippocampus [78] and lower metabolism in the medial temporal lobe, ascertained with fluorodeoxyglucose PET [79], than matched noncarriers. Consistent with the role of *APOE* ε4 in Aβ transport, postmortem studies suggest an association of *APOE* ε4 carrier status, amyloid pathology, and cognitive status [80, 81]. In vivo studies using amyloid imaging show that persons without the ε4 risk allele are more likely to lack demonstrable cerebral amyloid deposition [82], although this relationship is not always found [79, 83]. An association of *APOE* ε4 and the “AD pattern” of cerebral hypometabolism is more reproducible [84], and indeed appears to be present as early as the 20s or 30s in carriers [85]. This is evidence that the *APOE* ε4 risk allele may have effects on cerebral metabolism that are independent of its effects on Aβ metabolism. Furthermore, the demonstration of effects of *APOE* ε4 on brain morphology in infants suggests a role in development [86]. Recent studies found that a positive relationship between plasma and cortical Aβ could be demonstrated in persons lacking the ε4 allele but not in ε4 carriers [87], and that additional genetic variants contribute to AD risk and progression differentially depending on *APOE* genotype [88]. There is therefore substantial evidence that AD associated with the *APOE* ε4 allele differs in fundamental ways from other forms of AD.

## Implications for Treatment and Prevention

The implications of the diversity of AD for drug development are potentially tremendous and may in part account for the failure to date to develop effective disease-modifying therapeutics. Perhaps the most salient current example is the increased risk of intracerebral vasogenic edema and hemorrhage observed in relation to *APOE* ε4 status observed in a trial of the monoclonal antiamyloid antibody bapineuzumab [48•]. This is likely related to the presence of CAA, although this has not been definitively proven. Another example of how interventions may have differential effects on different genetic subtypes of AD is that of γ-secretase inhibitors. In vitro studies suggest that some mutant forms of presenilin are resistant to the effects of γ-secretase inhibitors [89]. Lack of efficacy of such a drug in a clinical study of ADAD therefore might not necessarily predict lack of efficacy in LOAD. ApoE-oriented therapeutics might also be expected to have differential effects and risks related to *APOE* genotype. The drug bexarotene, which increases ApoE levels, was found to rapidly clear amyloid plaques and improve cognition in a transgenic mouse model of AD [90]. As the effect was thought be mediated by native ApoE, one would expect a differential response depending on the recipient’s *APOE* genotype.

Clinical trials in persons at risk of genetic forms of AD provide a unique opportunity to intervene prior to the development of symptoms and therefore provide the possibility to prevent the disease. Such an approach is being implemented in the context of ADAD [91, 92], *APOE* ε4 homozygotes [92], and DS [93]. In the DIAN observational study of persons at risk of ADAD mutations, comprehensive clinical, imaging, chemical biomarker, genetic, and pathology data are being obtained, allowing the detailed characterization of a large ADAD population. This has demonstrated the sequential changes occurring in these measures during preclinical disease [12], informing the design and conduct of prevention studies in this same population [91]. For example, the verification that levels of Aß42 begin to decline in the CSF 25 years prior to the estimated onset of symptoms and cerebral deposition of amyloid can be detected 15 years prior to the estimated onset of symptoms made possible definition of the age range in which asymptomatic persons might be enrolled in a clinical trial with these biomarkers as outcomes [12]. The collection of data from persons with diverse ADAD mutations occurring in the DIAN observational study will also provide the opportunity to identify mutation-specific effects as has been done with regard to age of onset [11•]. The outcomes of similar approaches in *APOE* ε4 homozygotes and DS will help clarify the commonalities and differences among these subtypes of AD and ultimately inform the degree to which study results can be generalized.

## Conclusions

Although there appear to be commonalities in the pathways through which the AD phenotype originates, there also are differences that can be identified on the basis of their genetic origins. Treatment modalities should be chosen and trials designed with this in mind. Ideally, the different genetic causes should be specifically targeted with appropriate interventions. Lacking this, clinical trials should be pursued with the intention of taking into account in post hoc analyses known and possibly still unknown genetic factors.
